# A bumpy road ahead for genetic biocontainment

**DOI:** 10.1038/s41467-023-44531-1

**Published:** 2024-01-20

**Authors:** Dalton R. George, Mark Danciu, Peter W. Davenport, Matthew R. Lakin, James Chappell, Emma K. Frow

**Affiliations:** 1 School for the Future of Innovation in Society, Arizona State University, Tempe, AZ 85287 USA; 2 School of Biological & Health Systems Engineering, Arizona State University, Tempe, AZ 85287 USA; 3 School of Life Sciences, Arizona State University, Tempe, AZ 85287 USA; 4 Department of Computer Science, University of New Mexico, Albuquerque, NM 87131 USA; 5 Center for Biomedical Engineering, University of New Mexico, Albuquerque, NM 87131 USA; 6 Department of Chemical & Biological Engineering, University of New Mexico, Albuquerque, NM 87131 USA; 7 Department of Biosciences & Department of Bioengineering, Rice University, Houston, TX 77005 USA

**Keywords:** Policy, Biotechnology, Synthetic biology

## Abstract

While the research community continues to develop novel proposals for intrinsic biocontainment of genetically engineered organisms, translation to real-world deployment faces several challenges.

The environmental release of bioengineered organisms is increasingly being suggested for a variety of applications, including bioremediation^1^, biosequestration^2^, bio-mining^3^, environmental biosensing^4^ and conservation^5^. The objectives of many environmental release applications shift the goals of biocontainment from preventing organism spread outside of closed spaces (e.g., laboratories or bioreactors) to managing the persistence of engineered organisms and their genetic material in open, dynamic environments. In the scientific literature, discussions of environmental release are often accompanied by calls for robust “intrinsic biocontainment”^6^, where containment mechanisms are genetically engineered into the organism to limit and control its spread and persistence. A number of intrinsic biocontainment approaches have so far been proposed, and can be grouped into two overarching strategies (see Fig. 1). First, gene-flow barriers attempt to limit the spread of genetic material through lateral gene transfer, which refers to the ability of cells to directly exchange DNA molecules with one another or absorb DNA from external environmental sources. This can be accomplished through conditional lethality strategies (such as toxin anti-toxin systems^7^ or targeted DNA degradation^8^), or through limiting plasmid replication^9^ or deleting natural competence genes from target cells^10^. Second, strain/host control strategies seek to prevent survival and growth of engineered microbes outside specific environmental conditions using growth restriction and fitness control strategies such as metabolic auxotrophy^11^, kill switches^12^, and conditional essentiality^13^. Over the past decade, researchers and developers have expanded the technical toolkit of intrinsic biocontainment techniques, with new approaches using orthogonal sequences^14^, synthetic auxotrophy^15,16^, CRISPR-based kill switches^17^, sequence-entanglement^18^, and “cell-free” systems^14^.

**Fig. 1:**
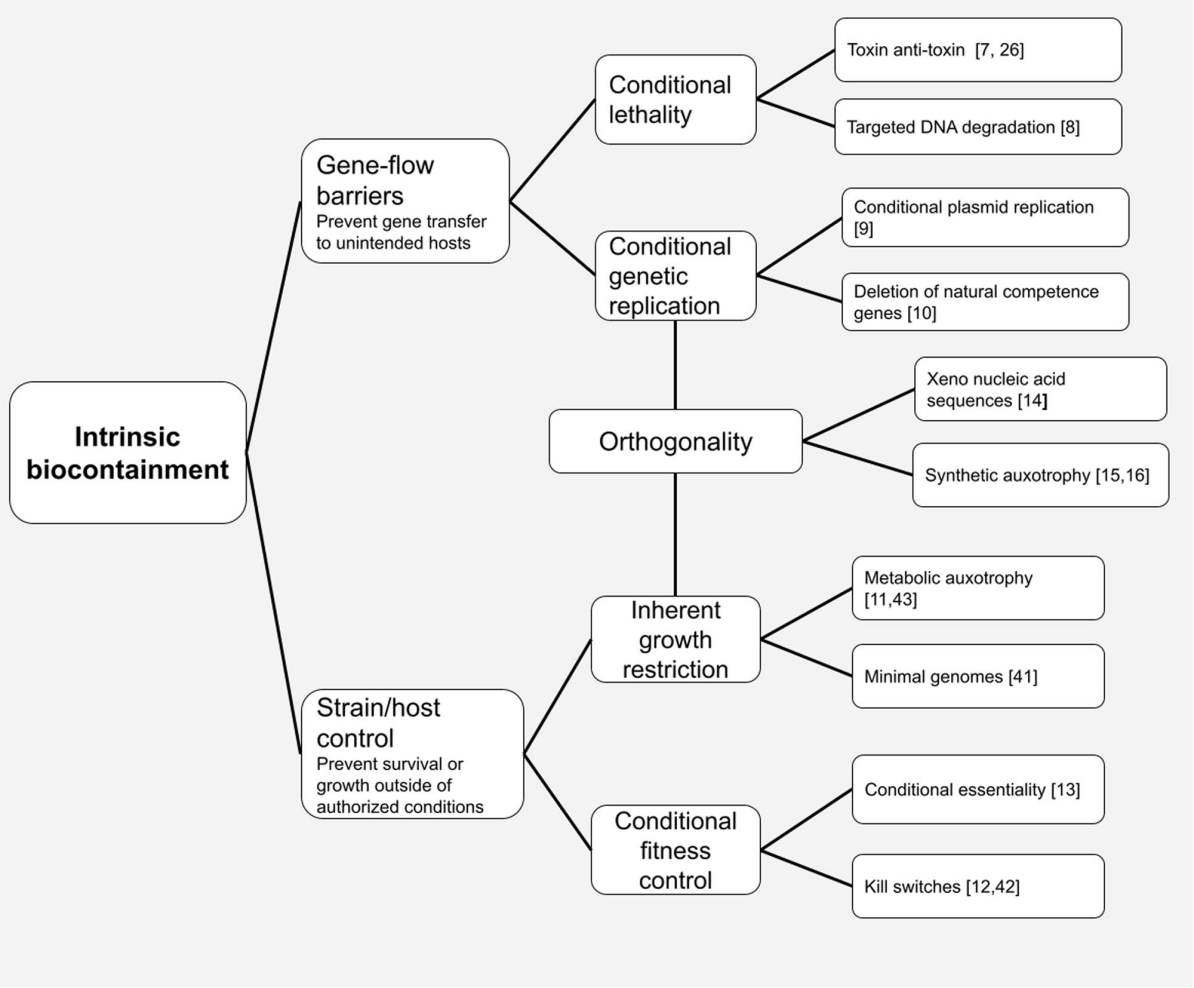
Intrinsic biocontainment technologies. This schematic presents a variety of intrinsic biocontainment technologies that have been proposed and/or developed, grouped according to different core strategies (e.g., preventing organism growth or limiting gene transfer). References (indicated in square brackets on the figure) offer illustrative examples of the indicated technology^7–16,26,41–43^. Note this figure and associated references are not intended to convey an exhaustive list of intrinsic biocontainment technologies; we anticipate them to evolve as additional biocontainment strategies and technologies are developed.

Yet in practice, very few biotechnology products deployed in real-world settings have thus far incorporated intrinsic biocontainment mechanisms. Here, we explore key challenges facing the implementation of engineered intrinsic biocontainment outside of closed environments. In addition to identifying challenges around testing capacity and regulatory uncertainty, we suggest expanding the framing of biocontainment to attend to issues beyond human and environmental safety risks. We offer some recommendations for action in the US and beyond.

## Laboratory research and testing challenges

The potential efficacy of intrinsic biocontainment technologies faces several uncertainties. First, there are limited test data and metrics available for evaluating efficacy in the laboratory. The most consistently used measurement is escape frequency, which quantifies the persistence of engineered microorganisms on non-permissive growth media. However, variations in what is considered acceptable detection limits, together with a lack of standardized test conditions for evaluating escape frequency across different environments, decreases the reliability of this metric^15,19^. The possibility of horizontal gene transfer from engineered organisms to wild-type organisms has also not been consistently tested in laboratory or field studies, therefore little is known about its risk potential.

Second, limited testing of intrinsic biocontainment mechanisms has been done under real-world conditions. The variety of possible real-world contexts (soils, ponds, oceans, etc.) and application purposes (bioremediation, biosensing, etc.) complicates the development of simple, standardized tests. Models can be used to simulate efficacy in real-world environments^20,21^, but testing methods developed in the laboratory are difficult and potentially costly to translate into complex, real-world environments. This may reinforce academic incentives that favor publishing high-profile, novel biocontainment proposals over developing more resource-intensive field trials and longitudinal evaluation studies of possible ecological effects^22^.

Furthermore, there is little capacity within US universities and their institutional biosafety committees (IBCs) to evaluate novel intrinsic biocontainment mechanisms. The primary charge for IBCs is to prevent accidental release of engineered organisms from contained environments^23^. This has led to a focus on ensuring appropriate physical containment measures (e.g., physical infrastructure including walls, floors and ceilings, biosafety cabinets, and personal protective equipment) to prevent exposure to engineered biological materials and hazards, and to control their spread in the case of accidental escape. The *NIH Guidelines for Research Involving Recombinant DNA or Synthetic Nucleic Acid Molecules*^23^ do include provisions for exempting host-vector systems with engineered “biological barriers” from physical containment requirements, but in practice the list of exempted systems is very limited and specific (Appendix E). Adding a new host-vector system to this list requires petitioning the NIH and providing comprehensive data justifying that the intrinsic biological containment mechanisms are sufficient (Appendix I-II-B). Importantly, the *NIH Guidelines* do not permit experiments involving the deliberate release of genetically engineered organisms into the environment without approval from the appropriate Federal agency (e.g., EPA, FDA, USDA). The administrative burdens involved in seeking exemption from physical biocontainment or securing approval for deliberate release discourage academic researchers and their IBCs from developing a robust knowledge base around the possibilities for intrinsic biocontainment mechanisms to stand alone as containment strategies.

## Designing biocontainment strategies for open environments

A broad and critically important challenge for researchers, developers and policy makers is to define what effective biocontainment means in different environmental contexts. There are currently no clear metrics for evaluating biocontainment “success” in open environments. Does it make sense to define successful biocontainment at zero unintentional spread of organisms and genetic material throughout an environment? Is some spread of genetic material tolerable up to a certain threshold, based on either concentration or environmental effects? Defining success is particularly challenging given the dearth of field research measuring the spread of genetically engineered materials in the environment and the lack of monitoring technologies and strategies for detecting the spread of genetic material in different environmental contexts^19,22^. Without broad consensus regarding metrics or capacity for long-term monitoring, the ability to demonstrate successful containment in field trials or environmental deployments will remain elusive.

What is clear is that biocontainment is not a one-size-fits-all approach to ensuring environmental biosafety. Researchers should consider several factors when thinking through different environmental applications of genetically engineered organisms^24^. First, the type of organism might influence the physical and genetic strategies that are most effective for controlling the spread of genetic material. For example, physical containment of an engineered rodent might be difficult given its mobility, but strategies that subdue or control sexual reproduction could be very effective at limiting the spread of genetic material^25^. In contrast, engineered bacteria might be easier to contain in physical space but are more susceptible to spreading genetic material through horizontal gene transfer. In the latter case, researchers and developers may prioritize strategies that privilege DNA degradation to limit horizontal gene transfer^26,27^.

Another ramification is that researchers and developers would benefit from thinking carefully about persistence across different dimensions—spatial, temporal, and ecological—and interactions between the organism and the environment over time. The size of the application area and desired time course of activity may vary across different application types and contexts. Possible considerations for future risk assessments could include weighing the level of exposure to risks such as pathogenicity, toxicity, competition with native species and/or horizontal gene transfer against the benefits of the proposed application of an engineered organism^28^. Ecological relationships might be minimally affected by the persistence of an engineered organism in the short term but have unforeseen impacts over time^29^. It is therefore important for researchers to consider how their proposed application could affect broader ecological dynamics in the long term, especially for proposals with larger effect areas and intended persistence in the environment.

## Challenges for industry adoption

### Regulatory uncertainty

Relatively few products that incorporate some form of engineered intrinsic biocontainment have to date been approved for field testing or commercialization by US regulatory agencies. Physical and naturally occurring biocontainment mechanisms—such as isolation distance, crop-topping, and self-pollination for GM crops—are far more commonly used. US regulatory agencies have together set little precedent for how intrinsic biocontainment fits into evaluations and assessments of bioengineered organisms. This regulatory uncertainty might lead to reluctance within industry to start incorporating elaborate intrinsic biocontainment mechanisms into novel organisms^30^, as opposed to relying on previously used physical or naturally occurring biocontainment approaches.

### Public controversy

A history of public controversy around intrinsic biocontainment technologies and genetically engineered crops may also have sensitized industry against pursuing novel approaches. One prominent example is the failure to commercialize genetic use restriction technologies (GURTs) in the late 1990s. GURTs used genetic modifications that restricted or eliminated the reproductive capacity of a crop, rendering the propagation of seed from that crop impossible^31^. While GURTs held promise towards the containment of transgenic materials in engineered crops, they were primarily promoted to protect intellectual property in foreign agricultural markets. This framing of GURTs contributed to a firestorm of public opposition. Monsanto’s “terminator seed” technology became a particularly hot target, with civil society organizations framing the technology as a violation of farmer rights to “save seed” and a threat to the food sovereignty of vulnerable populations^31^. A global moratorium on the commercialization of GURTs-based crops was enacted through the United Nations Convention on Biodiversity in 2000. This moratorium did not restrict longer-term research and development activities with GURT technology, but did halt any short-term commercial aspirations for GURTs-based crops at the time.

### Value proposition

Developing commercially viable genetically engineered organisms is a costly and time-intensive process for private firms^32^. Incorporating intrinsic containment into novel biotechnology products adds additional complexity and cost to the research and development processes. Coupled with regulatory uncertainty and concerns over potential social controversy, it is currently unclear whether and how intrinsic biocontainment offers added value or fits into industry development timelines for their product lines.

## Narrow framings of biocontainment

Genetic biocontainment strategies date back to the 1970s and were initially developed as a “technological fix”^33^ for preventing the escape and spread of engineered organisms and their biological materials within closed laboratory systems. Harms to human and environmental health were seen as the key risks to mitigate in this context, with physical containment measures becoming the standard approach to addressing exposure and escape.

Yet, what’s at stake for biocontainment changes into something more complex when genetically engineered organisms are explicitly designed to leave the lab and persist for longer periods of time and at larger scales in the environment^34^. By looking across a variety of historical containment ‘failures’ among environmentally released GMO products, we can observe a much broader set of risks and complex societal relationships emerge around biocontainment practices. For example, the failure to commercialize GURT technology (mentioned above) raises questions around the ownership and intellectual property dimensions of biocontainment. The large-scale physical containment failure of genetically modified Starlink corn in the late 1990s highlights important regulatory, economic and supply chain dimensions of biocontainment: contamination of US food supplies with Starlink (approved by US regulators as an animal feedstock but not for human consumption) resulted in recall of over 300 different food products and a 7% drop in the price of corn^35^. The regulatory and economic risks of biocontainment failures can also be international in scope, as when GM papaya seeds were found to have contaminated the organic papaya market in Hawaii in the 1990s, and Japanese importers began rejecting shipments^36^. None of these examples suggest widespread harm to human or environmental health resulting from biocontainment failure; rather, harm was realized in broader social, economic, and geopolitical terms. Such cases prompt us to think about a role for biocontainment in managing a much broader set of relationships outside the laboratory.

In keeping with these examples, public concerns around biotechnologies often extend beyond questions of health and safety. For example, questions relating to corporate concentration of economic and political power, inequalities in access and ownership, trust in regulatory bodies, and ethical concerns regarding the intrinsic value of life are routinely raised in public discussions of genetically modified organisms. There is a tendency within the research community to invoke biocontainment as a way to promote public trust^15,17,22^, but addressing the kinds of concerns raised in public debates about the control of engineered organisms takes more than assurance of human health and environmental safety.

Genetically engineered organisms introduced into the environment enter complex biological and social worlds. While it’s tempting to think of biocontainment as a straightforward technological fix, we suggest that more holistic evaluation of biocontainment proposals is needed. This would include attending to their legal, economic, regulatory, and social dimensions in addition to evaluating biosafety.

## Recommendations

We envision several opportunities to bolster capacity building and deepen the conversation around evaluation and deployment of biocontainment technologies in real-world settings. First, funders can dedicate more resources to efforts to develop rigorous protocols and standards for testing intrinsic biocontainment in laboratory and field trial settings. A few programs—such as the USDA’s Biotechnology Risk Assessment Grants program—are explicitly investing in biocontainment efforts, but more is needed. Alongside increased investment in developing biocontainment technologies, projects like the Department of Energy’s Secure Engineered Ecosystem Design provide support for building environmental monitoring and biosensing infrastructure that is crucial for tracing the spread of engineered microbes. Such infrastructure should be funded, developed, and maintained in tandem with biocontainment technologies. These projects could be strengthened by collaborations with researchers outside the engineering biology community—for example, from soil ecology and environmental toxicology—who have expertise assessing risk from an environmental perspective.

Second, training on intrinsic biocontainment could be made a requirement for institutional biosafety committees. This could provide greater institutional support and supplemental guidance for researchers developing synthetic organisms and testing different biocontainment strategies on microbes, plants, and animals.

Third, while many within the research community frame intrinsic biocontainment as desirable and important for future innovations, perspectives from policy practitioners, industry representatives, and members of the public are currently less visible. Initiatives that bring together stakeholders from different sectors are needed to think through the scientific challenges, regulatory uncertainties, and broader societal dimensions of intrinsic biocontainment. Such cross-sectoral dialogue and collaboration could build capacity around a suite of interrelated questions including:

- What standards should apply to testing of biocontainment strategies in laboratory and field settings? How should successful biocontainment be defined in different environmental contexts?
- How might regulatory agencies best evaluate intrinsic biocontainment features in genetically modified organisms?
- Would companies design intrinsic biocontainment features if there were clear and accessible regulatory pathways?
- Is there a role for public engagement in the design, implementation and regulation of genetic biocontainment strategies?

Finally, local communities should have a say in the design and management of environmental applications of genetically engineered organisms. Public engagement can complement the design and implementation of biocontainment strategies and help build trust between scientists, developers, and local communities. Researchers should be cautious about centering activities around promoting the safety prospects of biocontainment without also attending to broader social, economic, and legal dimensions of releasing engineered organisms into the environment. Iterative dialogue, collaboration, and practices to align values with local communities are more effective for building public trust than outreach focused on exclusively on safety and security^37^. Models for public engagement are increasingly being explored and implemented as part of environmental biotechnology projects^38–40^ but often struggle to secure sufficient funding and resources compared with other research activities. Projects looking to perform field releases of engineered organisms should create budgets for parallel engagement activities to complement the experimental research as far upstream as possible.

Until progress is made on the challenges we have described, the existing gap between innovative biocontainment proposals and successful real-world implementation of biocontainment strategies will likely persist. We hope this article galvanizes a broader conversation about how to concretely address that gap and approach the environmental release of engineered organisms in a safe and socially responsible manner.
